# Reshaping of the gastrointestinal microbiome alters atherosclerotic plaque inflammation resolution in mice

**DOI:** 10.1038/s41598-021-88479-y

**Published:** 2021-04-26

**Authors:** Michael S. Garshick, Cyrus Nikain, Michael Tawil, Stephanie Pena, Tessa J. Barrett, Benjamin G. Wu, Zhan Gao, Martin J. Blaser, Edward A. Fisher

**Affiliations:** 1 Center for the Prevention of Cardiovascular Disease, Department of Medicine, New York University School of Medicine, New York, USA; 2 Leon H. Charney Division of Cardiology, Department of Medicine, New York University School of Medicine, New York, USA; 3 Division of Pulmonary, Critical Care, and Sleep Medicine, Department of Medicine, New York University School of Medicine, New York, USA; 4 Division of Pulmonary and Critical Care, Veterans Affairs New York Harbor Healthcare System, New York, NY USA; 5 Center for Advanced Biotechnology and Medicine, Rutgers University, 679 Hoes Lane West, Piscataway, NJ 08854 USA; 6 Marc and Ruti Bell Vascular Biology Program, Cardiovascular Research Center, New York University Langone Health, New York, USA

**Keywords:** Microbial communities, Cardiology, Cardiovascular biology

## Abstract

Since alterations in the intestinal microbiota may induce systemic inflammation and polarization of macrophages to the M1 state, the microbiome role in atherosclerosis, an M1-driven disease, requires evaluation. We aimed to determine if antibiotic (Abx) induced alterations to the intestinal microbiota interferes with atherosclerotic plaque inflammation resolution after lipid-lowering in mice. Hyperlipidemic *Apoe*^−/−^ mice were fed a western diet to develop aortic atherosclerosis with aortas then transplanted into normolipidemic wild-type (WT) mice to model clinically aggressive lipid management and promote atherosclerosis inflammation resolution. Gut microbial composition pre and post-transplant was altered via an enteral antibiotic or not. Post aortic transplant, after Abx treatment, while plaque size did not differ, compared to *Apoe*^−/−^ mice, Abx^–^ WT recipient mice had a 32% reduction in CD68-expressing cells (p = 0.02) vs. a non-significant 12% reduction in Abx^+^ WT mice. A trend toward an M1 plaque CD68-expresing cell phenotype was noted in Abx^+^ mice. By 16S rRNA sequence analysis, the Abx^+^ mice had reduced alpha diversity and increased Firmicutes/Bacteroidetes relative abundance ratio with a correlation between gut Firmicutes abundance and plaque CD68-expressing cell content (p < 0.05). These results indicate that in a murine atherosclerotic plaque inflammation resolution model, antibiotic-induced microbiome perturbation may blunt the effectiveness of lipid-lowering to reduce the content of plaque inflammatory CD68-expressing cells.

## Introduction

The development of atherosclerosis involves an interplay between circulating lipoproteins, the arterial endothelium, and immune cell activation. One potential contributor to atherosclerosis not fully explored is the gut microbiota, a symbiotic community of organisms inhabiting the gastrointestinal tract^1,2^. Alterations in gut microbiota populations are associated with innate immune system activation and systemic pro-inflammatory responses. For example, macrophage toll—like receptors (TLRs) are activated by bacterial lipopolysaccharide (LPS) which may enter the systemic circulation from the gut driving an M1, pro-atherosclerotic state^3,4^. Gut-derived endotoxemia has also been linked to reduced expression of ABCA1/ABCG1, genes involved in cholesterol efflux and plaque regression^5^.

Gut microbiota alterations, such as diminished bacterial diversity and increased Firmicutes/Bacteroidetes ratio, induced by antibiotics including tylosin, have been associated with pro-inflammatory immune responses^6–9^. Consistent with a link between these gut microbiota changes and atherosclerosis, immunization against certain microbial taxa decrease the Firmicutes/Bacteroidetes ratio, reducing both atherosclerotic macrophage plaque content and circulating pro-inflammatory cytokines, while enhancing anti-inflammatory M2 macrophages^10^. Conversely, gut-derived metabolites such as trimethylamine N-oxide (TMAO) have been associated with atherosclerosis, thrombosis, and clinical cardiovascular disease (CVD)^11^. Taken together, these studies suggest a connection between the gut microbiome, systemic inflammation, macrophage activation, and atherosclerosis development.

Lowering of atherogenic apoB-containing lipoproteins is a cornerstone of atherosclerosis prevention. However, meta-analysis of lipid-lowering therapies show only 30% reduction in the secondary prevention of CVD^12^. In murine models assessing the impact of lipid lowering on atherosclerosis and inflammation resolution (e.g. reduction in CD68-expressing foam cells), plaques are not completely remodeled or reduced in size, even under optimal regression conditions^13^. Therefore, to improve the efficacy of cardiovascular prevention strategies, a better understanding of atherosclerosis inflammation resolution is needed.

To begin to define the relationship between atherosclerosis inflammation resolution and gut microbiome characteristics, we used an established murine model^14^ in which donor atherosclerotic aortic arches from hyperlipidemic *Apoe*^−/−^ mice are transplanted into the abdominal aorta of wild-type (WT) recipient mice; in the WT milieu, plaques regress^14^. To perturb the gut microbiota, the recipient mice were given an antibiotic or not (control), immediately before transplant and during the post-transplant period^15^. We found that in the setting of atherosclerotic plaque inflammation resolution over 5 days, compared with the no-treatment control group, the antibiotic-treated mice had more extensive CD68-expressing cell infiltrates within plaques and polarization towards an M1 state. The treated mice had reduced bacterial diversity and higher Firmicutes/Bacteroidetes ratios which correlated with the extent of impaired atherosclerosis inflammation resolution. These data highlight a potential role of the microbiome to influence atherosclerosis inflammation resolution that could be harnessed^16^ to improve CVD prevention strategies.

## Results

### Antibiotic administration alters the microbiome

**Figure 1:**
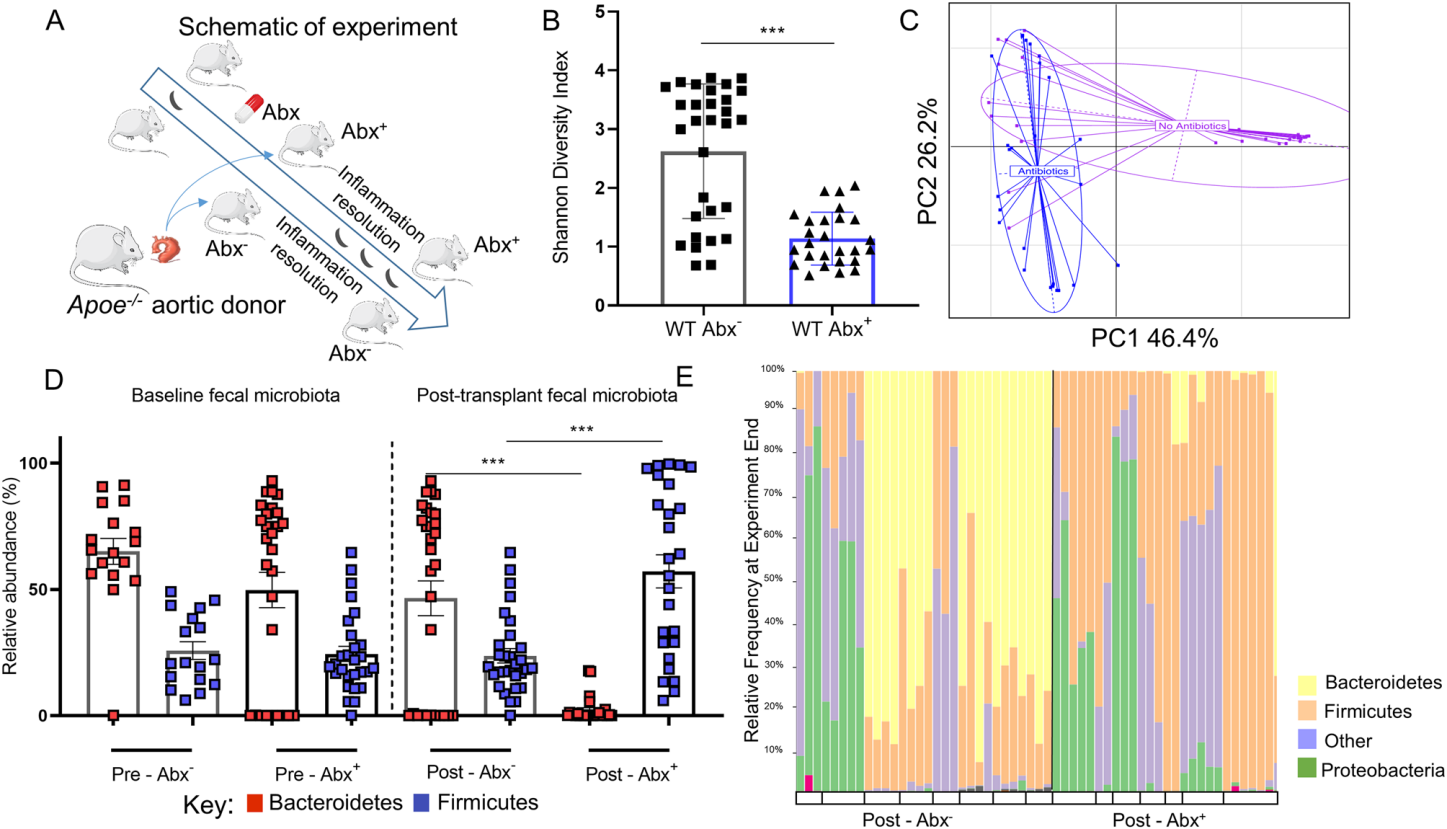
Alterations to gut microbiome in relation to exposure and inflammation. (**A**) Schematic of transplant experiments in which wild-type (WT) C57BL/6 mice received an aortic arch from an Apoe^−/−^ donor. One group of WT mice received the aortic arch transplant but were not given antibiotics (Abx^−^). The other group received antibiotic treatment 3 days prior to the aortic arch transplant, and throughout the 5 days post-transplant (Abx^+^). Fecal samples were collected from both groups pre- and post-aortic transplant. Analysis, comparing the antibiotic-exposed WT mice or not, according to: (**B**) Fecal samples post-aortic transplant; Shannon Diversity Index, and (**C**) weighted UniFrac analysis, to determine Beta-diversity; (**D**,**E**) Relative taxonomic abundance by phylum. In (**D**), each box represents a fecal sample while in (**E**), each box represents one mouse with bars indicating individual 16S rRNA fecal sample analysis. ABX^+^ mice had significantly reduced Bacteroidetes after treatment. (n =  ~ 7–8 mice/group) ***P < 0.001. Cartoons (1A) obtained from SMART Servier Medical Art under a Creative Commons Attribution 3.0 Unported License.

Tylosin is known to have sustained impact on the gastrointestinal microbiome including reductions in alpha diversity and increased Firmicutes/Bacteroidetes ratio^15^. To understand the contribution of the tylosin-induced microbiome alterations to atherosclerosis inflammation resolution, we employed a murine aortic transplant model of atherosclerosis inflammation resolution^17^. To ensure that the microbiome was perturbed prior to the arch transplant, one-half of the group of arch-recipient WT mice received tylosin starting 3 days prior to the transplant, and the exposure was continued until sacrifice on day 5 (Fig. 1A), and microbiota compositions analyzed.

At baseline, murine fecal composition in both WT Abx^−^ and WT Abx^+^ mice had similar bacterial compositions (beta-diversity, Supplemental Figure IA) with similar Firmicutes/Bacteroidetes ratios (Fig. 1D). As expected, in untreated (Abx−) mice post-transplant we noted relatively minor gut microbial changes (Supplemental Figure IB) with significant decreases in only relative *Bifidobacterium* and *Lactobacillus* composition. This suggests that aortic transplantation does not lead to a substantial shift in the gut microbiome.

After antibiotic administration, there was a significant decrease in gut microbial bacterial richness (Fig. 1B, p < 0.001). Analysis of community structure (beta-diversity), displayed a marked shift in the Abx+ group (Fig. 1B,C), which included a substantial increase in the Firmicutes/Bacteroidetes ratio (Fig. 1D) and significantly differed from the Abx− group (Fig. 1E). At lower taxonomic levels, post-aortic transplantation and antibiotics, Bacteroides were significantly reduced (Supplemental Figure IC). Comparison between post-transplant between Abx+ and Abx− mice showed reduced representation of taxa including Anaeroplasma, Lactobacillus, and Bacteroides, and increased representation of Turicibacter and Paenibacillus after antibiotic administration (Supplemental Figure ID). These results highlight that, as expected, tylosin causes substantial gut microbiome perturbation.

### Antibiotics delay atherosclerosis inflammation resolution

**Figure 2:**
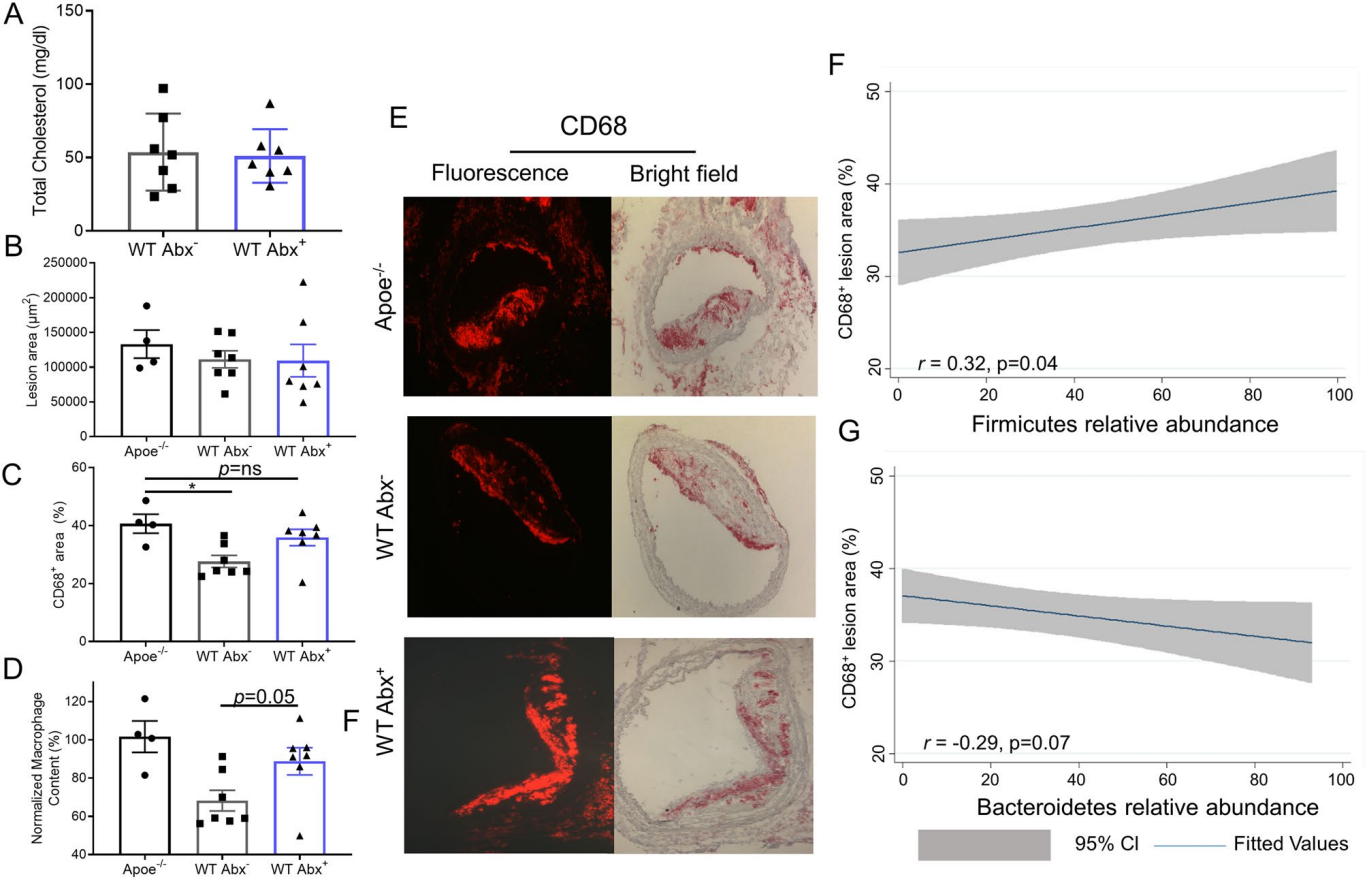
Antibiotic exposure impairs atherosclerotic plaque inflammation resolution. Analysis of aortic arch plaques from *Apoe*^*−/−*^ mice after 16 weeks on a Western Diet in WT-recipient mice 5-days post-transplant. Quantification of (**A**) Total cholesterol, (**B**) Lesion area, (**C**) percent CD68 + area within plaque, (**D**) CD68^+^ area normalized with respect to the *Apoe*^*−/−*^ donor mice. (**E**) Representative sections of aortic transplant arches from *Apoe*^*−/−*^, WT-post-Abx^+^ and WT-post-Abx^−^ groups, stained with anti-CD68 antibody at ×100 magnification, with bright field images shown for full morphology. Relation of CD68 + lesion areas with relative (**F**) Firmicutes and (**G**) Bacteroidetes taxa abundances of fecal samples taken at the experiment end. Mann–Whitney and Pearson’s correlation test used. (n = 4–7 mice/group) * < 0.05.

The transplantation of atherosclerotic aortic arches into *C57BL/6J* (WT) mice receiving a regular chow diet, results in predictable inflammation resolution of atherosclerosis, including loss of macrophages (e.g. loss of CD68-expressing foam cells) from within these plaques within 5 days^14,18^. On post-transplant day 5, there was no difference in total serum cholesterol between the Abx^−^ and Abx^+^ groups (Fig. 2A). The total plaque area also was not significantly different post-transplant between the *Apoe*^*−/−*^, WT Abx^−^, and WT Abx^+^ groups of mice (Fig. 2B). Next, we assessed the inflammatory and stability characteristics of the plaques (Fig. 2E). Compared to the *Apoe*^*−/−*^ mice at baseline, there was a 32% reduction in CD68-positive plaque area in WT Abx^−^ mice (p = 0.02) and a 16% reduction in necrotic core (p = 0.06, Supplemental Figure IIA) as expected. However, there was a 12% (non-significant) reduction in CD68-positive plaque area and 7% (non-significant, Supplemental Figure IIA) reduction in necrotic core in the WT Abx^+^ mice (Fig. 2C). The percent of collagen within plaque also trended lower in the WT Abx^+^ mice (4%) compared to the WT Abx^−^ mice (12%, p = 0.11, Supplemental Figure IIB). Finally, when normalized to the size of the *Apoe*^*−/−*^ CD68-positive plaque area, the WT Abx^−^ mice had 68% CD68-positive staining compared to 89% for WT Abx^+^ (p = 0.05, Fig. 2D).

**Figure 3:**
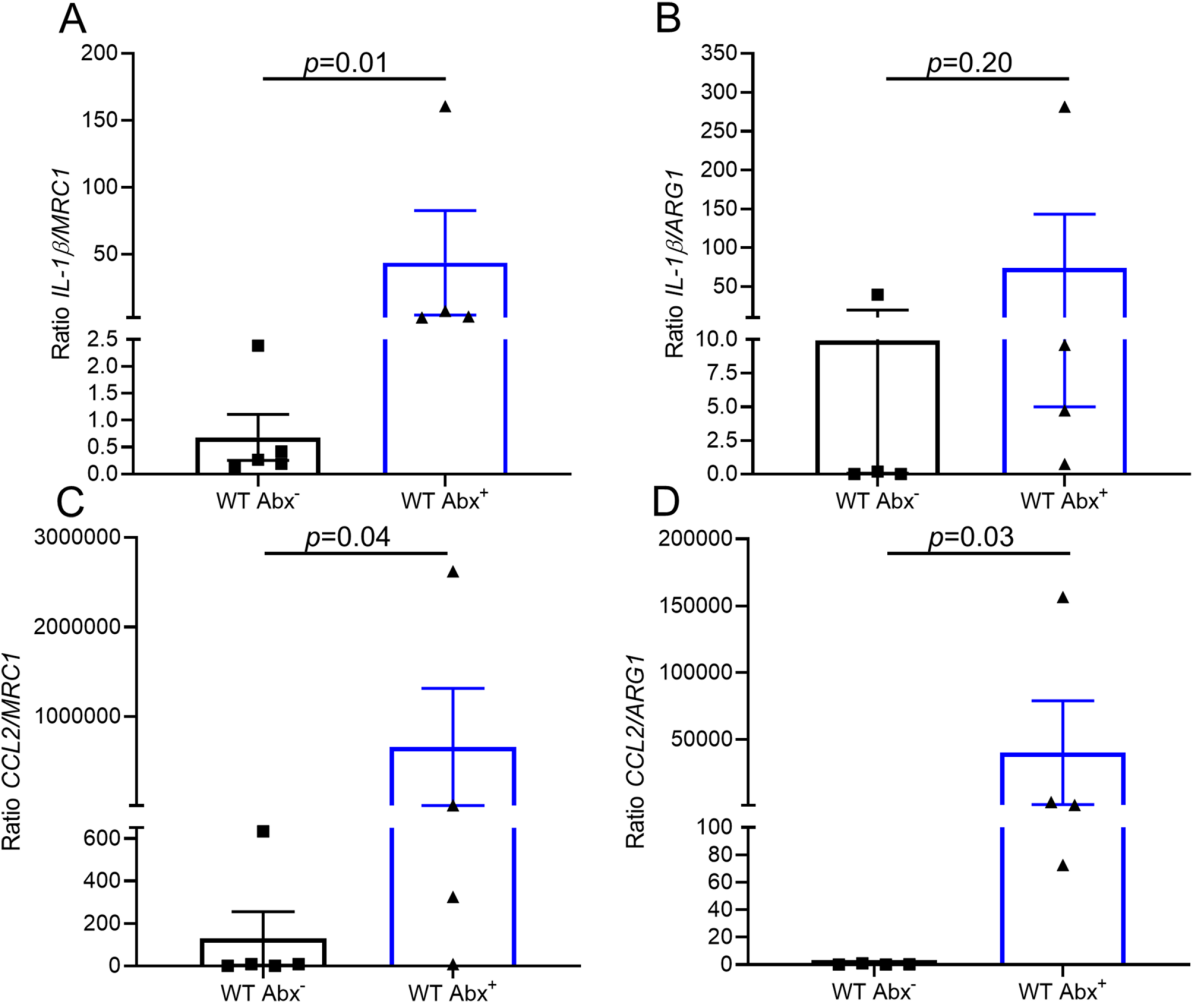
Antibiotic exposure alters CD68-expressing foam cells to an M1 state. Transcript expression via laser capture microdissection of CD68-expressing cells from atherosclerotic aortic arches in the WT post-Abx^+^ and WT post-Abx^−^ groups. (**A**) Ratio of CD68-expressing cell transcript expression of IL-1B/MRC1 (mannose receptor C-type 1), (**B**) IL-1B/ARG1 (arginase-1), (**C**) CCL2/MRC1, (**D**) CCL2/ARG1. Mann–Whitney test used; (n = 4–5 mice/group).

To further assess the CD68-expressing cellular phenotype, gene expression analysis using RNA obtained by LCM of plaque CD68-expressing cells was assessed. A bias toward inflammatory M1 polarization in WT mice that received antibiotics was seen, including higher IL-1β/MRC1, CCl2/MRC1, CCL2/ARG1 ratios and a trend toward higher IL-1β/ARG1 ratios compared to the WT mice that did not receive any antibiotic, which had more anti-inflammatory M2-like polarization features (Fig. 3). In summary, these results indicate that in a murine model of atherosclerosis inflammation resolution, antibiotic exposure impairs the loss of CD68-expressing foam cells from plaques, as well as altering them to an M1 state, with these changes independent of circulating cholesterol levels.

### A dysbiotic microbiome is directly related to CD68-expressing cell content within plaque

Finally, to understand how the microbiome findings relate to atherosclerotic plaque inflammation resolution, we examined the relationship between CD68-positive plaque area and relative abundance of Firmicutes and Bacteroidetes. For fecal samples obtained at the end of the experiment, a direct correlation of CD68+ plaque area with Firmicutes (*r* = 0.32, p < 0.05) was observed, but was inversed for Bacteroidetes (Fig. 2F,G). In the aggregate, our findings indicate that the antibiotic tylosin alters the microbiome to a dysbiotic state, delaying expected plaque inflammation resolution in terms of CD68-expressing cell content and polarization to an M1 phenotype, with an association noted between relative Firmicutes abundance and higher CD68-expressing cell plaque content.

## Discussion

A link between the gut microbiome and the pathogenesis of atherosclerosis has been hypothesized to occur through several mechanisms including endotoxemia-induced chronic inflammation, differential effects of gut microbial derived short-chain fatty acids, and production of the metabolite TMAO^4,11^. These factors, as well as changes in microbial compositions, have been previously associated with atherosclerosis progression^19^. Atherosclerosis develops early in life in western countries with adults harboring an appreciable plaque burden^20^. As such, investigating atherosclerosis inflammation resolution has the potential to substantially impact and improve upon cardiovascular prevention strategies^21,22^. In this study, we show that antibiotic administration leads to microbial alterations characterized by loss of microbial diversity, higher Firmicutes/Bacteriodetes ratio, and enhanced CD68-expresing foam cell (presumed to be macrophages) plaque content with M1 polarization. These findings suggest a pathway in which the antibiotic administration, by affecting the composition and activity of the microbiome delays inflammation resolution within atherosclerotic plaques.

Most bacteria within the human and mouse intestinal microbiome are members of the phyla Firmicutes and Bacteroidetes^23^. In this study, the macrolide antibiotic tylosin, which is predominantly used in veterinary medicine, was used to alter the microbial composition into a dysbiotic state, as we have shown in other murine studies^15,24^. Increases in the abundance of gut Firmicutes and in gut microbiota-derived LPS (endotoxemia) are independently implicated in macrophage M1 activation and pro-atherosclerotic clinical conditions including obesity and hypertension. Microbiota changes are also associated with enhanced TMAO production, itself a pro-atherosclerotic and pro-thrombotic metabolite^25–28^.

Furthermore, decreasing the Firmicutes/Bacteroidetes ratio is shown to reduce macrophage plaque content while enhancing polarization towards anti-inflammatory M2 macrophages^10^. Therefore, as expected in control (Abx^−^) mice post-transplant, we observed plaque inflammation resolution and compositional changes including CD68-expressing cell loss from within plaques and a bias towards M2 polarization, but not changes in plaque size, consistent with prior studies^17^. In lipid lowering environments in mice, the resolution of atherosclerotic plaques is promoted by recruitment of monocytes that have an M2 phenotype^18^. Our study, showing that tylosin administration impairs inflammation resolution, while sustaining CD68-expressing foam cells M1 polarization, suggesting that this process may be interrupted via gut microbial changes.

Prior studies support the likelihood that it was the microbiota changes and not tylosin which led to the impaired atherosclerosis plaque inflammation resolution^29^. Macrolide antibiotics such as tylosin (used in animal models), or such as azithromycin which are used therapeutically in humans, are considered to have anti-inflammatory properties per se^30^, but these are in the opposite direction from the observations in this model. We studied tylosin since prior data indicated that exposure leads to a sustained alpha diversity loss, with impact on Firmicutes/Bacteroidetes ratios, which are strongly associated with cardiometabolic changes in both human and animal models^15,31^.

Our study has other limitations such as the small sample size and that CD68 staining (main outcome measure), while the preferred method for identifying plaque macrophages, is not 100% specific for plaque macrophages and may also indicate vascular smooth muscle cells that acquire macrophage like features. We therefore assessed other markers of plaque stability including necrotic core and collagen staining to highlight compositional differences between antibiotic treated and untreated mice undergoing atherosclerotic inflammation resolution. Finally, we intended this study to establish whether there was sufficient reason to warrant additional mechanistic investigations required to further characterize effects on plaque macrophages and to distinguish the direct tylosin effects vs. those due to gut microbial alterations. Additional investigations, will therefore include larger study groups.

Those with advanced atherosclerosis and clinical CVD represent the group at highest risk for a cardiovascular event^32^. In randomized controlled trials investigating aggressive lipid lowering therapy (with statins), the residual risk of a cardiovascular event remains in excess of 50%^33^. Our findings on the link between gut microbial features and impaired atherosclerosis inflammation resolution after aggressive lipid lowering suggests one mechanism why this may occur. A multitude of factors may impact the gut microbiota including diet, weight, and pharmacologic agents^34^. Therefore, given the noted interaction between the microbiome and atherosclerosis progression, and now plaque inflammation resolution, future studies may be warranted to investigate the clinical impact of microbiota adjustment in the prevention of secondary CVD events.

## Conclusions

In conclusion, in a murine model of atherosclerosis inflammation resolution, antibiotic administration that reduced diversity and shifted the Firmicutes/Bacteroidetes balance reduced the impact of aggressive lipid lowering on atherosclerotic plaque inflammation resolution. These findings suggest a role of the microbiome in the biology of atherosclerosis regression, and possibly in the development of the disorder. Further research is required to confirm and expand on these findings.

## Materials and methods

### Experimental design

All murine studies were performed in accordance with the recommendations in the NIH Guide for the Care and Use of Laboratory Animals and Institutional Animal Care and Use Committee of the New York University School of Medicine reviewed and approved protocols^35^. Our group has described in depth the aortic transplantation models^14,18^. Briefly, hyperlipidemia female *Apoe*^−/−^ mice were weaned at 4 weeks of age and fed a standard high-fat western diet (WD; 21% fat, 0.3% cholesterol) for 16 weeks to develop complex atherosclerotic aortic arch plaques, then sacrificed. The aortic arch was harvested, and transplanted into the abdominal aorta of 20-week old recipient *BC57BL/6J* (WT) mice, maintained on standard chow diet. To assess plaque regression and CD68-expressing cells (presumed to be plaque macrophages) efflux out of plaques, 5-days later^18^, the recipient mice were sacrificed and tissues and intestinal contents analyzed. As a plaque progression control, other *Apoe*^−/−^ mice were fed a WD and received an aortic arch transplant at the same age and conditions as the WT mice, before their sacrifice 5 days later and tissue analyses.

To perturb the microbiota in a subset of the recipient mice, the macrolide antibiotic tylosin was added to drinking water at 0.333 mg/ml corresponding to 50 mg/kg/day based on an estimated daily water consumption of 150 ml/kg of body mass, as described^7,15^. The recipient mice received tylosin pre-transplant for 3 days, and then for 5 days post-transplant until sacrifice^7,15^. Plasma total cholesterol levels were performed through enzymatic assays (Wako Life Sciences). All assays were performed per manufacturer’s recommendations. Fecal samples were collected pre-transplant and daily through sacrifice on post-transplant day 5 and stored at − 80 °C.

### Gut microbiota sample processing and microbiota analysis

Mouse fecal microbiota DNA was extracted using the PowerSoil-htp 96 Well Soil DNA Isolation Kit (MoBio, Carlsbad CA, USA). The V4 region of bacterial 16S rRNA genes was amplified in triplicate reactions using barcoded fusion primers 515F/806R, which amplifies bacterial and archaeal 16S genes^36,37^. The DNA concentration of the V4 amplicons for each sample was measured using the Quant-iT PicoGreen dsDNA assay kit (ThermoFisher Scientific, Waltham MA, USA). Samples were pooled in equal quantities. These set pools were then purified using the Qiaquick PCR purification kit (Qiagen, Hilden, Germany) to remove primers, quantified using the high-sensitivity dsDNA assay kit and the Qubit 2.0 Fluorometer (Life Technologies, Eugene OR, USA) and then combined at equal concentrations to form the sequencing library. The V4 region of ~ 254 bp was sequenced using the Ilumina MiSeq 2 × 150 bp platform at New York University Langone Medical Center (NYULMC) Genome Technology Center.

### Fecal 16S microbiome analysis

16S rRNA gene sequences were demultiplexed and analyzed utilizing the Quantitative Insights into Microbial Ecology (QIIME version 2.0 2019.01 release) pipeline for analysis of microbiome data (Bolyen PEERJ/Mature). QIIME2 output was then uploaded in R version 3.5.1 (R Core Team [2017]. R: A language and environment for statistical computing. R Foundation for Statistical Computing, Vienna, Austria. [https://www.R-project.org]) utilizing QIIME2R v0.99 [https://github.com/jbisanz/qiime2R]). Phylogeny was assigned utilizing DADA2^38^. Differences in microbial alpha diversity were calculated using the Shannon Diversity Index^39^ and differences in beta diversity determined using weighted UniFrac^40^.

### Plaque analysis

The grafted aortic arches were removed after 5 days, after perfusion with saline containing 10% sucrose, bifurcated, embedded in OCT, and frozen. Serial sections (6 μm thick) were cut using a cryostat and mounted on glass slides, with 12 sections per slide. Cluster of differentiation 68 (CD68)+ (a marker of monocyte-derived) cells were detected and quantitated as described^18,41^. Briefly, sections were fixed and permeabilized with 100% ice-cold acetone, blocked with rabbit serum, stained with anti-CD68 antibody (rat anti-mouse CD68 antibody, Bio-Rad, Hercules CA), biotinylated anti-rat IgG secondary antibody (Vector Laboratories, Burlingame CA), and visualized using the Vectastain ABC kit (Vector Laboratories). Slides were counterstained with hematoxylin/eosin (H&E, Sigma), dehydrated in an ethanol gradient and xylene (Fisher Scientific, Waltham NH), and mounted with coverslips using Permount (Fisher Scientific). Bright-field images of the stained aortic arch sections were imaged using a DM4000B (Leica-Camera, Wetzlar, Germany) at 10× magnification, with a fluorescent lamp (Prior Scientific Instruments Ltd, Cambridge, UK) to visualize the Vecta Red-stained macrophage (CD68-expressing cells) plaque content; on average, 10 slides were stained and imaged/aortic arch. Morphometric analyses to determine plaque area, macrophage (CD68-expressing cells) composition and percent macrophages (CD68-expressing cells) within plaque were performed with ImagePro Plus 7.0 software (Media Cybernetics). The plaque area was determined by outlining cell layers between the endothelium and smooth muscle cell layers using the counterstained bright-field image, and the fluorescent image was then overlaid on the bright-field image to determine the macrophage plaque content^42^.

Plaque collagen content and necrotic core size were assessed in aortic arch cross-sections using Picrosirius red-stained arterial sections. Briefly, formalin fixed slides were washed in PBS, and stained with Picrosirius red solution (Polysciences, Inc, PA) for 1 h. These slides were then washed, dehydrated with ethanol and xylene, and mounted with coverslips. Images were obtained under bright field (to identify plaque) and polarizing light with a Keyence BZ-X810 (Keyence, Osaka, Japan). The images were analyzed using ImageJ software [National Institutes of Health (NIH), Bethesda, Maryland, USA] by a trained person blinded to the experimental conditions. Necrotic core was defined as the areas in which the extracellular matrix was lacking and replaced by dead cells and cellular debris or nothing at all. The amount of collagen was defined as the percent of positive Picrosirius red staining within the total plaque area, all as previously described^43,44^.

### Laser capture microdissection (LCM) and real-time quantitative PCR analysis

All procedures were as previously described^45^. During serial sectioning, additional aortic arch sections were collected using polyethylene naphthalene (PEN) membranes for eventual laser-capture microdissection. PEN membrane slides were subjected to H&E staining for morphology and tissue visualization, then the PEN membrane slides were loaded onto a Leica LMD 6500 LCM system using sample-matching CD68-stained slides to guide selection of plaque macrophages (CD68-expressing cells). LCM sections were collected in an RNAse-free tube containing extraction buffer. PicoPure RNA Isolation Kit (Molecular Devices, San Jose CA) was used to isolate RNA from the LCM collected sections. RNA quality and quantity were measured using the Agilent RNA 6000 Pico Chip (Agilent, Santa Clara CA) system. The RNA samples were amplified and converted to cDNA using QuantiTect Whole Transcriptome Kit (Quiagen, Germantown MD), then RT-qPCR was performed on QuantStudio 7 Flex (ThermoFisher Scientific) using TaqMan probes (ThermoFisher Scientific) and primers for Il-1β and the M2 markers mannose receptor C-type 1 (MRC1) and arginase-1 (ARG1). A relative increase in IL-1β expression signified polarization to an M1 pro-atherosclerotic state^46^.

### Statistical analysis

Data are reported as mean ± SEM. Normality was assessed using the Shapiro–Wilk test for normality. Independent two-sided t-tests were used for normally distributed data while the Mann–Whitney U test was used for data with a non-normal distribution. Pearson’s correlation coefficient was used to evaluate the association between two variables. All analyses were performed in GraphPad Prism 8.2.0 or Stata V. 14 (College Station, Tx: StataCorp LP). A *P* < 0.05 was considered significant.

## Supplementary Information

Supplementary Information.

## Abbreviations

CVD: Cardiovascular disease
TLR: Toll-like receptors
LPS: Lipopolysaccharide
TMAO: Trimethylamine N-oxide
WT: Wild-type
WD: Western diet
CD68: Cluster of differentiation
LCM: Laser capture microdissection
PEN: Polyethylene naphthalene
MRC1: Mannose receptor C-type
ARG1: Arginase-1
