# Supplementary material for: Reshaping of the gastrointestinal microbiome alters atherosclerotic plaque inflammation resolution in mice
Source: Sci Rep. 2021 Apr 26;11:8966. doi: 10.1038/s41598-021-88479-y (PMC8076321; doi:10.1038/s41598-021-88479-y)
Supplement: Supplementary file 1 — Supplementary Information. [file 41598_2021_88479_MOESM1_ESM.docx]

**Reshaping of the Gastrointestinal Microbiome Alters Atherosclerotic Plaque Resolution in Mice**

Michael S. Garshick^1,2^, MD, MS, Cyrus Nikain^2,^ MSc, Michael Tawil^2^, BA, Stephanie Pena^2^, MS, Tessa J. Barrett^2^, PhD, Benjamin G. Wu^3,4^, MD, MS, Zhan Gao^5^, MD, Martin J. Blaser^5^*, MD, Edward A. Fisher^1,2^*, MD, PhD

^1^Center for the Prevention of Cardiovascular Disease, Department of Medicine, New York University School of Medicine; ^2^Leon H. Charney Division of Cardiology, Department of Medicine, New York University School of Medicine; ^3^Division of Pulmonary, Critical Care, and Sleep Medicine, Department of Medicine, New York University School of Medicine; ^4^Division of Pulmonary and Critical Care, Veterans Affairs New York Harbor Healthcare System, New York, NY; ^5^Center for Advanced Biotechnology and Medicine, Rutgers University, Piscataway, NJ.

*Co-senior author


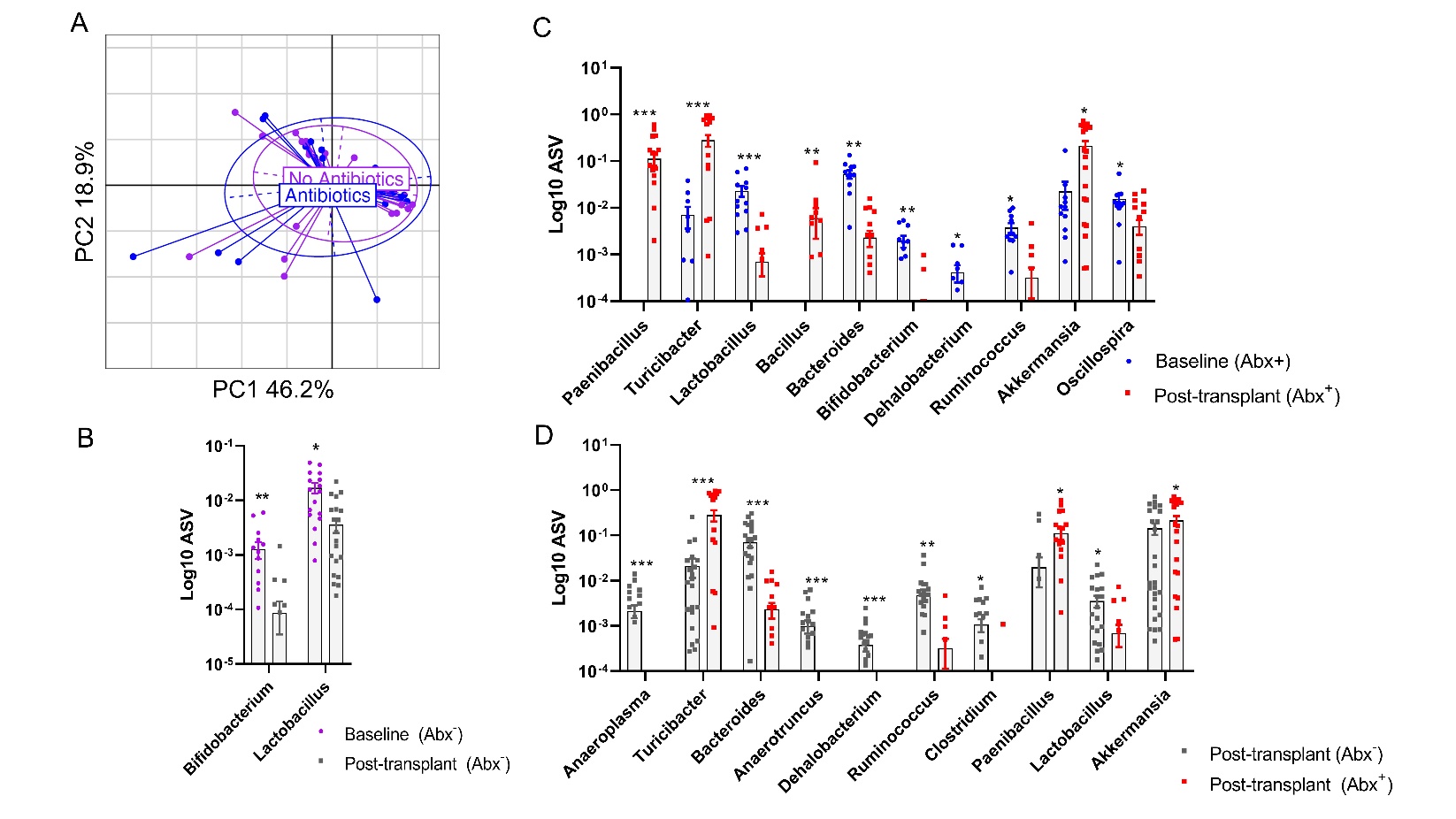
**Supplemental Figures and Figure Legends**

**Supplemental Figure I. Gut microbiome compositional changes. (A)** UniFrac analysis of pre-transplant, pre-antibiotic fecal pellets from mice that will be exposed to antibiotics (Abx^+^) or not (Abx^-^). Taxa level changes in baseline fecal samples and post-aortic transplant in **(B)** mice that did not receive antibiotics (Abx^-^) and **(C)** mice that did receive antibiotics (Abx**^+^). (D)** Comparison of post-transplant fecal microbiome composition at the taxa level in Abx^+^ and Abx^‑^ mice. ASV; Amplicon sequence variant (from 16S rRNA fecal sample analysis). n=4-5 mice/group. *<0.05, **<0.01, ***<0.001


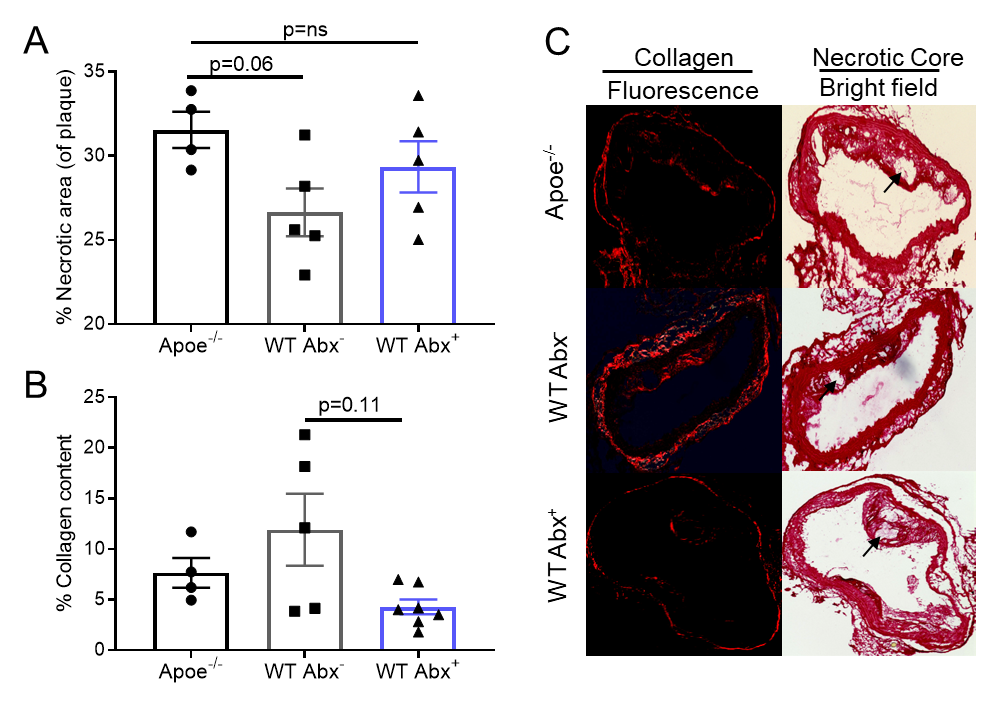


**Supplement Figure II. Antibiotic exposure suggests alterations to necrotic core and collagen formation.** Analysis of aortic arch plaques from *Apoe^-/-^* mice after 16 weeks on a Western Diet in WT-recipient mice 5-days post-transplant. Quantitation of **(A)** percent of necrotic core and **(B)** percent collagen within total plaque area. **(C)** Representative sections of aortic transplant arches from *Apoe^-/-^*, WT-post- Abx^+^ and WT-post- Abx^-^ groups, at x100 magnification. Necrotic core defined as the percent area of plaque where extracellular matrix is lacking (black arrows). Collagen content defined as the percent of the plaque positive for Picrosirius red staining (using fluorescence microscopy). Mann-Whitney test used. (n=4-7 mice/group).
